# Intraarticular gold microparticles using hyaluronic acid as the carrier for hip osteoarthritis. A 2-year follow-up pilot study

**DOI:** 10.1038/s41598-024-77760-5

**Published:** 2024-11-01

**Authors:** Sten Rasmussen, Emilie Skjoldemose, Nia Kristine Jørgensen

**Affiliations:** 1 Department of Clinical Medicine, Aalborg University, 249 Selma Lagerløfs Vej, room 11.03.025, DK-9260 Aalborg, Gistrup, Denmark; 2 Department of orthopedic surgery, Aalborg University Hospital, Aalborg, Denmark

**Keywords:** Osteoarthritis, Hip, Gold microparticles, Hyaluronic acid, Outcomes research, Medical research, Rheumatology, Disability, Osteoarthritis

## Abstract

We recently conducted a pilot study in which we discovered that introducing metallic gold microparticles into the knee joint can help reduce pain caused by osteoarthritis for up to two years. Additionally, we identified significant proteomic changes in the synovial fluid and serum within eight weeks of the procedure. In this study, we aimed to evaluate whether there may be a clinical relevant effect of intra-articular injection of gold microparticles on hip osteoarthritis. A cohort of 22 patients, aged ≥ 18 years, with pain ≥ 3 months, and Kellgren-Lawrence OA grade 2–4, were included. Metallic gold 20 mg, 72.000 pieces, 20–40 µ-meter BerlockMicroImplants (BMI) were injected into the hip joint using hyaluronic acid as the carrier. In total, we treated 26 hip joints. The primary outcome was the Western Ontario and McMaster Universities Arthritis Index (WOMAC). WOMAC pain decreased from 11 (2–20 to 3 (0–8), stiffness from 6 (0–8) to 1 (0–4), and activity from 43 (18–68) to 11 (0–27), all *P* = 0.0001. When adjusting for the minimally relevant differences, the P-values were 0.0015 for WOMAC pain, 0.26 for stiffness, and 0.011 for activity. Combined intraarticular treatment with metallic gold microparticles and hyaluronic acid may improve hip joint pain and function. Joint stiffness did not improve when assessed against the minimal clinically relevant difference. This study suggests a basis for a future placebo-controlled randomized trial of gold microparticles and hyaluronic acid in hip osteoarthritic patients.

**Supplementary Information:**

The online version contains supplementary material available at 10.1038/s41598-024-77760-5.

## Introduction

Hip osteoarthritis (OA) is a prevalent degenerative joint disorder affecting millions of people worldwide^1^. The disease’s impact on patients’ daily lives necessitates effective and safe treatment modalities. Despite differences in pathogenesis and response to treatment, the management of hip osteoarthritis is often based on results from the treatment of knee osteoarthritis^1^. In recent years, intra-articular treatments have gained prominence as viable therapeutic options. The intra-articular therapies have gained attention due to their localized action, potentially reducing systemic side effects commonly associated with conventional treatments^2^.

These therapies involve intra-articular medications into the affected joint, offering targeted relief while minimizing systemic side effects^3^. In hip OA, intra-articular therapies primarily aim to reduce pain, improve joint function, and enhance the overall quality of life for affected individuals. Several types of intra-articular medications are utilized for hip OA, each with its unique mechanisms of action and benefits^4^. However, the risk of septic arthritis is a concern^4^.

Corticosteroids are powerful anti-inflammatory agents commonly used in intra-articular injections. They help reduce inflammation within the joint, alleviating pain and improving mobility. Corticosteroid injections do provide short to medium-term relief for patients with hip OA, making them a popular choice, especially in acute exacerbations of symptoms^5,6^.

Visco supplementation involves injecting hyaluronic acid (HA) derivatives into the joint. HA is a naturally occurring substance in synovial fluid that lubricates and cushions the joint. In patients with hip OA, the viscosity and elasticity of synovial fluid are reduced. Viscosupplementation aims to restore these properties, thereby reducing friction, relieving pain, and improving joint function. While the evidence for viscosupplementation in hip OA is not as robust as in knee OA, some studies suggest potential benefits, especially in specific patient populations^7^.

Platelet-rich plasma (PRP) therapy involves collecting a small amount of the patient’s blood, processing it to a concentration of platelets, and injecting it into the joint. Platelets contain growth factors that may promote tissue healing and repair. Although research on PRP for hip OA is ongoing, preliminary studies show low to moderate results in terms of pain reduction and functional improvement^8^.

Stem cell therapy may be promising for osteoarthritis. However, for knee osteoarthritis, there are several methodological flaws in the meta-analysis of clinical studies on knee osteoarthritis, and it is not possible to perform a sound meta-analysis^9,10^. For hip osteoarthritis, there is limited data^11^.

Intra-articular therapies, including corticosteroid injections, viscosupplementation, PRP injections, and regenerative medicine therapies, offer various levels of pain relief and functional improvement for patients with hip OA. However, the evidence supporting these interventions varies in quality and quantity^1–12^.

Research in this field continues to advance. Healthcare providers must stay updated on the latest findings and consider individual patient factors when choosing the most appropriate intra-articular therapy for hip OA management. A network meta-analysis of 10 studies with relatively small sample sizes finds that compared with placebo, no intraarticular injections demonstrate a potentially significant difference at up to 6 months for patients with hip osteoarthritis and conclude saline is as effective as corticosteroids, hyaluronic acids, and platelet-rich plasma^12^.

According to the existing literature, there is a lack of knowledge on whether to treat hip osteoarthritis (OA) with an injection, or not. The management of hip OA is generally based on the results obtained from the treatment of knee OA. In a pilot study of intra-articular metallic gold microparticles for knee OA^13^, we used the patient’s own synovial fluid as the carrier. In another group of patients with hip osteoarthritis we administered intra-articular gold microparticles and HA as a standard procedure. The objective of this study is to determine if intra-articular gold microparticles and HA have a role to play in the management of hip OA. We hypothesize that the use of intra-articular gold microparticles and HA can improve hip OA symptoms and function.

Building upon previous research emphasizing the importance of targeted approaches in hip OA management^14–16^ and the inhibition of gold on inflammation^17,18^, this study aims to explore whether there may be a clinically relevant effect of intraarticular gold microparticles and HA for hip OA.

## Methods

In this open uncontrolled pilot study, we evaluated clinical function and pain at baseline and eight weeks after intra-articular injection of gold microparticles and HA for hip OA. We measured the stability of pain and function at a two-year follow-up.

### Study group

The patients received gold microparticles, 20 mg sterile 99.99%, a total of 72.000 particles, 20–40 μm in diameter (BerlockMicroImplants (BMI), Berlock ApS)^13,19^ injections into the hip joint guided by ultrasound. Specifically, two milliliters of hyaluronic acid (Suplasyn^®^ 20 mg/ml) were mixed with the sterile gold microparticles and injected into the patient’s hip.

### Patients

**Fig. 1:**
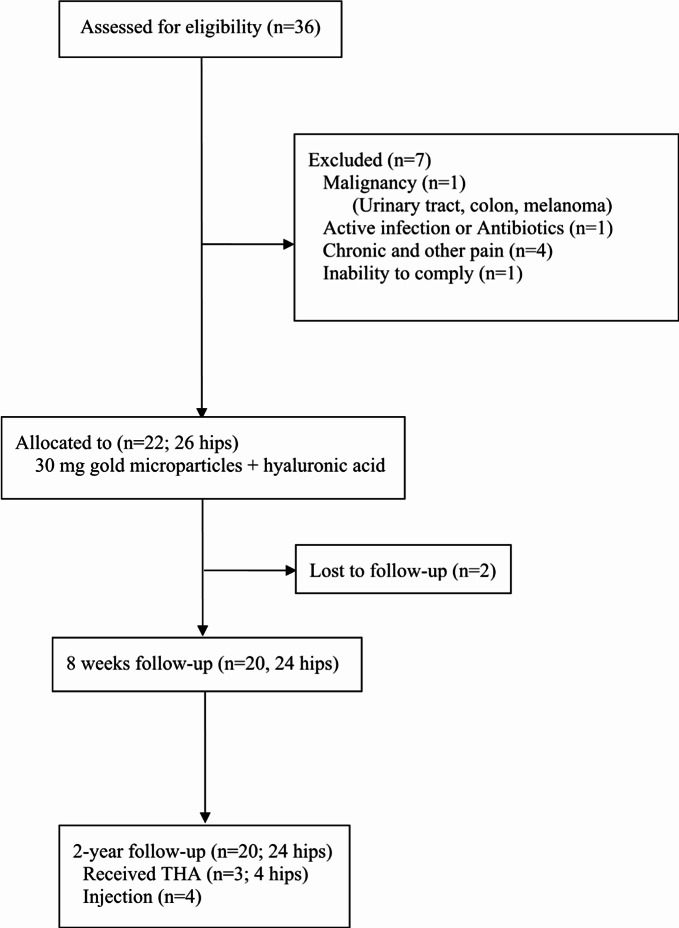
Patient flow chart. Enrollment, treatment, and follow-up.

From January 2019 through 2021, patients with radiographically confirmed moderate hip OA (Kellgren-Lawrence grade ≥ 2), pain for more than three months, and maximal pain intensity VAS (Visual Analogue Scale, 0–10) ≥ 5 during the last week were enrolled at the Department of Orthopedic Surgery, Aalborg University Hospital, Denmark. The exclusion criteria were (1) malignancy, (2) active infection and antibiotic treatment, (3) active treatment with steroids, biological or other anti-rheumatic medication, (4) history of chronic pain condition, (5) inability to comply with the protocol, and (6) inadequacy in written and spoken national language (Fig. 1).

### Main outcome variable

The primary patient-reported outcome measure pre-treatment and at 8 weeks and 2-year follow-up was the Western Ontario and McMaster Universities Arthritis Index (WOMAC) sub-scores for pain, stiffness, and function, containing 24 questions: five pain questions, two stiffness questions, and 17 physical function questions. Each question utilizes a 5-point scale, from 0 (none) to 4 (extreme)^20^. In the analysis, we used a minimal relevant difference of 6 points for WOMAC pain, 2 for WOMAC stiffness, and 17 for WOMAC function^21,22^.

### Other variables

The secondary outcome measure PainDetect questionnaire (PDQ)^23^ comprises three major components: gradation of pain, pain course pattern, and radiating pain. Seven questions evaluate the gradation of pain. The patient scored each question using a 0 to 5 score with 0 = never, 1 = hardly noticed, 2 = slightly, 3 = moderately, 4 = strongly, and 5 = very strongly. There is one question evaluating pain course patterns. Patients select from one of four pictures indicating which pattern best describes their course of pain. Each picture has a unique score of 0, -1, or + 1 (two pictures with two different patterns have the score + 1). One question evaluates radiating pain with a yes (score of + 2) or no (score of 0) response option. PDQ is scored from 0 to 38, with total scores < 13 considered to represent nociceptive pain, 13–18 possible neuropathic pain, and > 18 representing neuropathic pain.

Using the Global Rating of Change Scale^24^ we asked the question, *“Concerning your hip*,* how will you describe yourself compared to immediately before the injection of gold into your knee?”* and evaluated the answer on an 11-point scale from very much worse (-5) to completely recovered^5^ with a score of “0” indicating no changes.

### Procedures

The Consort guideline for reporting non-randomized pilot and feasibility studies was followed^25^. All authors take responsibility for the integrity and accuracy of the reported data and the credibility of the study to the protocol.

The regional data protection agency approved the project by 12/12/2023 (K2023-136).

The study followed the principles of the Declaration of Helsinki. According to the Danish Center for Ethics, health science questionnaire surveys and interview surveys that do not include human biological material are not subject to notification according to the Danish Ethics Committee Act (Sect. 14, subsection 2 of the Committee Act, no 1338 01/09/2020).

All participants gave informed consent to participation in the research via written forms and verbally.

### Statistical analysis

The Stata software, version 18.0 (StataCorp) was used when analyzing the clinical outcomes. Baseline characteristics are presented in median and range, as well as mean and standard deviation. Patient-reported data and results are ordinal and are analyzed using non-parametric Wilcoxon’s sign test used for all measurements. When adjusting for the minimal clinically relevant difference, the improvement in stiffness is adjusted with 17, improvement in function with 4, and improvement in pain with 6 before analyzing using Wilcoxon’s sign test.

Data generated during the current study are available within the paper, in the supplement and from the authors.

## Results

A total of 22 patients were enrolled, and four patients received treatment of both hips. Table 1 presents the baseline characteristics. At 8 weeks follow-up, two patients were lost. The other 20 patients completed the follow-up. Within the first year, four hips in three patients needed a re-injection with gold and one hip injection with steroids. Between 1 and 2 years of follow-up, two patients with Kellgren-Lawrence score III-IV needed total hip arthroplasty, one received total hip arthroplasty of both gold-treated hips, and one received a hip arthroplasty. All 20 patients were included in the analysis.

**Table 1:** Baseline characteristics of 22 patients and 26 hips.

Female/Male sex	15/7
Age – year	71 (46–88); 70 (48.1–91.8)
BMI	25 (19–42); 27 (13.9–39.8)
Kellgren-Lawrence Score, 24 hips	
II	5
III	19
IV	2
Womac scores	
Pain	11 (2–10); 11 (3.9–19.0)
Stiffness	6 (0–8); 5 (0.72–10.2)
Function	46 (18–68); 42 (12.9–71.7)
Pain detect	8 (1–15); 8 (0.06–16.7)
Cardiovascular disease	7
Diabetes	3
Neurological disease	2
Inflammatory Bowel Disease	1

Values are median and range; and mean and 95% CI. Scores on the Kellgren-Lawrence scale range from 0 to 4, with scores of 2, 3, and 4 indicating definite osteoarthritis and higher scores indicating more severe disease.

**Fig. 2:**
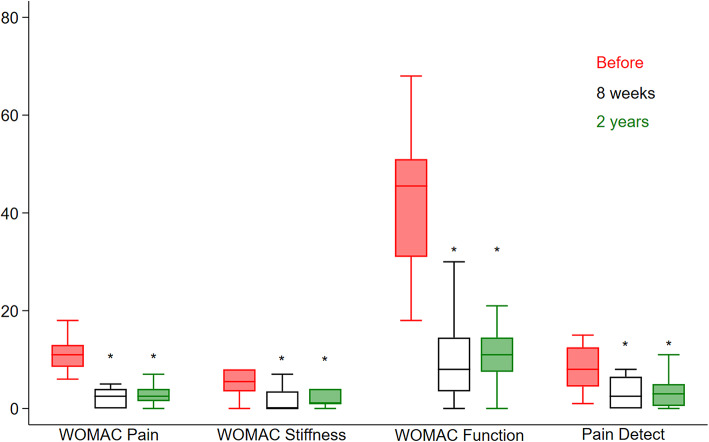
Results. WOMAC pain, stiffness, and activity, and PainDetect, before treatment, and 8 weeks and 2 years after intra-articular injection of 20 mg gold micro-particles and 2 mL hyaluronic acid in 22 hip OA patients. Median and quartiles. * Represents significance compared to before treatment.

Compared to the baseline, the three WOMAC sub-scores^20^ and PDQ^23^ all improved at two years of follow-up (Fig. 2). WOMAC pain decreased from 11 (6–20) to 3 (0–8), stiffness from 6 (0–8) to 1 (0–4), and activity from 46 (18–68) to 11 (0–27), all *P* < 0.0001. When adjusting for the minimally relevant differences, the P-values were 0.0015 for WOMAC pain, 0.021 for stiffness, and 0.041 for activity. PDQ decreased from 8 (1–16) to 3 (0–11) (*P* = 0.007).

There was no difference in WOMAC sub-scores from 8 weeks to 2-year follow-up.

The PDQ^23^ scoring found that 6/20 of the patients had nociceptive pain with a score ≥ 13 before treatment, and no patients had nociceptive pain two years after.

On the 11-point Global Rating of Change Scale (23.), at the 2-year follow-up, 17 patients reported a positive effect, one no effect, and two were worse ((3 (-3;5))). There was no difference in the Global Rating of Change Scale from 8 weeks to 2-year follow-up.

## Discussion

This exploratory, open, proof-of-concept study on patients with painful hip osteoarthritis showed that intra-articular combined treatment with gold microparticles and HA may provide pain relief after eight weeks and continuous clinical benefits throughout the two years of follow-up. HA was the carrier for the injection of the gold microparticles. Whether or not intra-articular HA has an effect^6^, it may peak at eight weeks and persist for at least six months^26^ and may influence the initial results of this study. Gold does have an anti-inflammatory effect in different settings^18^. From a historical point of view, gold gained this therapeutical effect serendipitously best expressed in rheumatoid arthritis, other autoimmune diseases, and in skin disorders^12,18^. A long anti-inflammatory effect of gold microparticles observed in knee osteoarthritis^14^ may, in this study, explain the continuous clinical effect throughout the two years of follow-up.

The prevalence of several types of osteoarthritis through 2050, is continuing to rise and will lead to a substantial increase in case numbers because of population growth and aging and because there is no effective cure for osteoarthritis^27^. By 2050, the estimated case numbers will increase by 60–100%, with BMI being an important modifiable risk factor. Clinical guidelines recommend including patient exercise, education, and weight management in combination with Non-Steroidal Anti-Inflammatory Drugs and intra-articular steroid injections^28^. There is a lack of consensus on the pharmacological options and the use of adjunctive treatments that is a challenge for adherence to the guidelines. The future guidelines need to provide implementation guidance and consider applicability.

There is a constantly evolving pharmaceutical management of osteoarthritis^29^. New different and promising treatments are emerging, and conventional medicines (e.g., acetaminophen and NSAID’s) are becoming less acceptable due to concerns with efficacy and safety. The latest evidence and recommendations need to be considered to make an informed decision when optimizing the treatment plans for patients with osteoarthritis.

There is a call for other and new treatments that can relieve the inflammation that plays a significant role in the degeneration of the joint^30^. The present study in hip osteoarthritis, combined with a previous study in knee osteoarthritis^13^, suggests that gold microparticles may be a new targeted approach in osteoarthritis.

Several studies indicate that OA is not only an aging process, but also an inflammation-related disease where synovitis facilitates the pathogenesis of OA^31^, and the synovitis plays a significant role in the genesis of pain^17,32^. In a previous study in gold microparticles for knee OA^14^, we found biomarkers indicating a change in inflammation^17^,

As an open pilot study with no control group the results must be tested and evaluated in a randomized study. The HA may have influenced the results in the early follow-up up to six months^6,26^. We did choose the suplasyn HA for its low to medium molecular weight to keep the gold microparticles floating, not falling as in saline, and to be able to deliver the gold microparticles into the joint. In addition, suplasyn HA is degraded leaving the gold microparticles as the sole active agent. The injection by itself, the placebo effect, and the intention to treat may influence and improve the results. In addition, it is known for hip and knee OA that injection of normal saline for up to six months yields a statistically significant and clinical meaningful improvement^13,33^. For the primary outcome WOMAC, using point estimation, not converting to 0-100 scores, and adjusting for minimal clinically important improvement strengthen the interpretation of the results^21,22^. In addition, the intervention was carefully standardized and administrated by the same physician (SR), the pain evaluation before and after eight weeks was done by SR and research assistant GG, and the 2-year follow-up by two medical students (ES and NKJ).

In conclusion, intraarticular treatment with metallic gold microparticles and hyaluronic acid may improve hip joint pain and function. Joint stiffness did not improve when adjusting for the minimal clinically relevant difference. Intraarticular gold microparticles and hyaluronic acid relieved pain and enhanced function in 17 of 20 patients. This study suggests a basis for a future placebo-controlled randomized trial of gold microparticles and hyaluronic acid in hip osteoarthritic patients.

### Data sharing statement

All data supporting the findings of this study are available within the paper and its Supplementary Information.

## Electronic Supplementary Material

Below is the link to the electronic supplementary material.

Supplementary Material 1

## Data Availability

All data supporting the findings of this study are available within the paper and its Supplementary Information.
